# It's a matter of time: Reframing the development of cognitive control as a modification of the brain's temporal dynamics

**DOI:** 10.1016/j.dcn.2015.08.006

**Published:** 2015-09-13

**Authors:** R. Matthew Hutchison, J. Bruce Morton

**Affiliations:** a Center for Brain Science, Harvard University, Cambridge, MA, USA; b Department of Psychology, University of Western Ontario, London, Ontario, Canada

**Keywords:** Cognitive control network, Dynamics, fMRI, Functional connectivity, Resting-state

## Abstract

Cognitive control is a process that unfolds over time and regulates thought and action in the service of achieving goals and managing unanticipated challenges. Prevailing accounts attribute the protracted development of this mental process to incremental changes in the functional organization of a cognitive control network. Here, we challenge the notion that cognitive control is linked to a topologically static network, and argue that the capacity to manage unanticipated challenges and its development should instead be characterized in terms of inter-regional functional coupling dynamics. Ongoing changes in temporal coupling have long represented a fundamental pillar in both empirical and theoretical-based accounts of brain function, but have been largely ignored by traditional neuroimaging methods that assume a fixed functional architecture. There is, however, a growing recognition of the importance of temporal coupling dynamics for brain function, and this has led to rapid innovations in analytic methods. Results in this new frontier of neuroimaging suggest that time-varying changes in connectivity strength and direction exist at the large scale and further, that network patterns, like cognitive control process themselves, are transient and dynamic.

## 1 Introduction

Cognitive control – the capacity to consciously adapt thought and action in the face of unanticipated challenge – follows a protracted developmental trajectory (Diamond, 2013). Like many developing intellectual skills, cognitive control is a robust longitudinal predictor of intellectual, social, and health-related outcomes (Moffitt et al., 2011). What makes cognitive control unique among intellectual skills is that it deals with exceptions – computational challenges for which there are no single, ready-made solutions. Almost by definition then, the development of cognitive control must be linked to an emerging ability to flexibly explore alternative configurations of a problem space. A prominent view, built on theoretical and empirical foundations (Johnson, 2001), links the development of cognitive control to age-related changes within a distributed set of linked cortical and subcortical regions collectively referred to as the cognitive control network (CCN) (Cole and Schneider, 2007, Dwyer et al., 2014, Fair et al., 2007). But what is occurring across the CCN to enable cognitive flexibility and what changes in the brain, either functional or structural, are linked to the development of cognitive control?

The present perspective argues that cognitive control should not be reduced to a fixed topology that is incrementally optimized over development. Instead, we suggest that cognitive control can be reframed as an ongoing and dynamic interplay of distributed regions (including those outside the traditional CCN) whose temporal features, (“chronnectome”; Calhoun et al., 2014) are modified as a function of age. We first introduce the CCN and its study in relation to development – empirical investigations dominated by functional magnetic resonance imaging (fMRI) approaches. We argue that although previous studies provide unprecedented insight into developmental changes in brain organization, they do not adequately capture brain activity that unfolds at the shorter timescales in which cognitive control is actually realized. Dynamic approaches that consider time-varying changes in functional connectivity (FC) and initial explorations using this framework are then discussed before outlining questions that deserve continued exploration.

## 2 The cognitive control network over development

### 2.1 Cognitive control network defined

**Fig. 1:**
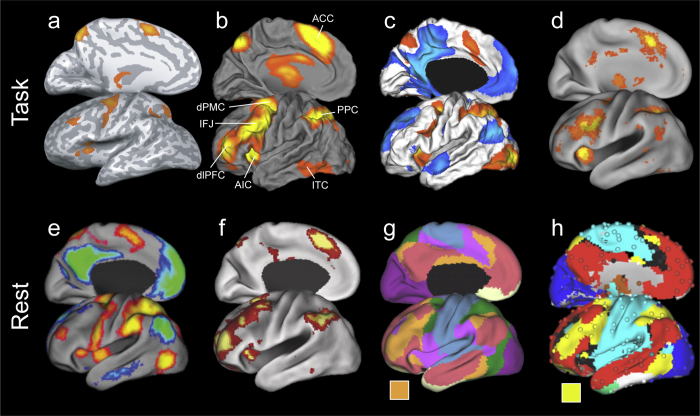
Maps of the cognitive control network derived using rest (top row) and task-based (bottom row) functional imaging approaches. Images are taken from Cole and Schneider (2007) (a), Satterthwaite et al. (2013) (b), Dwyer et al. (2014) (red–yellow, c), a forward inference meta-analysis using the using the Neurosynth platform (www.neurosynth.org) with a search term ‘cognitive control’ (d), Fox et al. (2005) (red–yellow, e), Vincent et al. (2008) (f), Yeo et al. (2011) (orange, g), Power et al. (2011) (yellow, h). Abbrev.: ACC, anterior cingulate cortex; AIC, anterior insular cortex; dlPFC, dorsal lateral prefrontal cortex; dPMC, dorsal premotor cortex; IFG, inferior frontal junction; ITC, infero-temporal cortex; PPC, posterior parietal cortex.

The CCN can be defined as a structurally and functionally distinct set of cortical and subcortical brain regions that is linked to the capacity for exerting control (for similar definition, see Cole and Schneider, 2007), where the term “network” indicates a collection of items with pairwise temporal relationships (for discussion, see Power et al., 2010). Constituent regions include selected parts of frontal (dorsolateral prefrontal, inferior frontal junction, dorsal premotor), insular (anterior insula), cingulate (anterior cingulate cortex), temporal (infero-temporal cortex), and parietal (posterior parietal cortex) cortex (see Fig. 1), as well as thalamic nuclei and the basal ganglia. While convergent with what Fox et al. refer to as the task-positive network (Fox et al., 2005), this definition is admittedly broad, and encompasses what is likely a family of cognitive control networks. Indeed several whole-brain parcellation schemes subdivide the CCN (Cole and Schneider, 2007) or task-positive network (Fox et al., 2005) into a number of structurally and functionally distinct subnetworks, variously termed: (1) fronto-parietal, dorsal attention, and ventral attention networks (see Yeo et al., 2011; 7-network parcellation); (2) fronto-parietal task control, dorsal attention, and ventral attention networks (see Power et al., 2011; graph-based parcellation); (3) cingulo-opercular task-set maintenance and fronto-parietal moment-to-moment adjustment networks (Dosenbach et al., 2007); (4) executive control and dorsal visual stream components (Beckmann et al., 2005); and (5) salience and executive control networks (Seeley et al., 2007). While acknowledging the importance of subdividing the CCN, points of contrast between static and dynamics approaches to FC that will be made in this discussion remain true whether the CCN is defined broadly or as a family of subnetworks. Therefore, in the interest of economy, we will use the term CCN to refer to this distributed set of regions.

### 2.2 The CCN and its development

Questions concerning its precise demarcation notwithstanding, there is a general consensus that the CCN is a stable feature of the human connectome, important for cognitive control, and subject to developmental change. These ideas rest largely on three related lines of evidence: (1) task-based fMRI activation studies; (2) resting-state fMRI (rsfMRI) FC studies of intra-network connectivity; and (3) task-based and rsfMRI studies of inter-network connectivity, especially those focused on interactions between the CCN and the default network (DN).

#### 2.2.1 CCN: evidence from task-based activation studies

Task-based fMRI activation studies provide consistent evidence that almost all regions of the CCN are more active when demands on cognitive control are high as compared to when they are low (Fig. 1, top row). How these profiles of activity change with age is less clear (for review, see Crone and Dahl, 2012). Some studies report age-related increases in activity, consistent with the idea that children engage cognitive control processes more robustly as they develop, whereas other studies demonstrate age-related decreases in activation, suggesting, perhaps, that the CCN functions more efficiently over time. Firm conclusions concerning the importance of age must, however, be drawn with caution in light of age-correlated differences in task performance. Indeed, inter-individual variability in task performance controlled for age is a much more robust predictor of CCN activity than age controlled for differences in performance (Satterthwaite et al., 2013). These issues notwithstanding, regions comprising the CCN readily show correlated increases in activity as demands in cognitive control increase.

#### 2.2.2 CCN: evidence from rsfMRI studies of intra-network connectivity

A persuasive source of evidence concerning the existence of the CCN comes rsFC analysis (e.g., Vincent et al., 2008; Yeo et al., 2011, Power et al., 2011, Spreng et al., 2013). The method is based on the finding that regions that co-activate in association with task administration also exhibit correlated intrinsic BOLD activity in the absence of an explicit task (Biswal et al., 1995; for review see Fox and Raichle, 2007). In early work examining the CCN with a rsfMRI approach, Fox et al. (2005) extracted spontaneous BOLD time courses from three regions, the intra-parietal sulcus, the frontal eye fields, and the middle temporal region and correlated these with time courses from every other voxel encompassing the brain (a seed-based approach). The resulting map showed a set of regions whose time courses correlated positively with each of the seed-regions and was highly convergent with maps of the CCN generated using task-based techniques (Fig. 1e). This has since been replicated across multiple resting-state studies including those that parcellate the entire cortex (see Fig. 1, bottom). Note that there is some ambiguity in defining the CCN at rest because of the absence of an explicit task to guide both the analysis and the interpretation of the resulting functional map. This is exacerbated by the fact that CCN sub-networks are less self-integrated and self-contained than other functional networks, with sparse within sub-network connections and more between sub-network connections (Baker et al., 2014, Spreng et al., 2013, Vincent et al., 2007) that can cause forced-membership approaches to assign nodes to different resting-state networks.

Graphical representation and analysis of rsMRI data has also become a highly influential means of characterizing developmental changes in brain networks generally, and the CCN in particular. Although real developmental changes may be obscured by age-correlated motion artifact (see Power et al., 2012; Satterthwaite et al., 2012; Van Dijk et al., 2012; Yan et al., 2013), there appears to be converging support for several general principles. First, short-distance connections between anatomically proximal regions tend to decrease in strength over development whereas long-distance connections between anatomically more distal regions tend to increase in strength over development (Fair et al., 2007). Second, edges that increase in strength over development typically connect nodes that are functionally connected in adults, such as nodes within either the adult CCN or the adult DN (Power et al., 2010). These findings are preliminary and sensitive to methodological decisions such as thresholds and choice of seeds. However, they imply that developmental change is topological in nature: parts of what will become complex networks – such as the CCN – in adulthood, are in children, weakly connected to each other, but moderately connected to regions that will ultimately become parts of other networks.

#### 2.2.3 CCN: evidence from rsfMRI studies of inter-network connectivity

Task-based and rsfMRI studies of functional interactions between the CCN and other networks – most especially the DN – represent a third body of evidence supporting the existence and functional specialization of the CCN. Tasks that impose substantial demands on cognitive control are associated with activation in CCN and deactivation in the DN, with the extent of activation and deactivation within the CCN and the DN, respectively associated with higher in-scanner task performance, and higher offline measure of executive functioning (Satterthwaite et al., 2013). Regions that deactivate in association with administration of cognitive control tasks are highly overlapping with maps of regions whose intrinsic activity negatively correlates with the CCN. Anti-correlations are strongest between selected sub-networks of the CCN – in particular dorsal and ventral attention networks – and the DN, but in general the pattern holds across the network. These observations are an important point in the argument that the CCN is anatomically and functionally unique as it suggests that the CCN (or a significant portion of its subcomponents) instantiates control through competitive interaction with the DN whose function is decidedly non-executive.

Task-based studies of development suggest a compelling extension of this general story, in that there is evidence to indicate that the degree of DN deactivation during cognitive control tasks is less pronounced among children as compared to older individuals (Luna et al., 2010, Marsh et al., 2006). Whether this difference is a bona-fide developmental difference or an age-correlated performance difference is unclear, as the degree of DN deactivation during cognitive control task performance is robustly associated with performance after controlling for age, but unrelated to age after controlling for differences in performance (Satterthwaite et al., 2013). Resting-state studies are important in this regard insofar as they eliminate the possibility that age-differences reflect differences in explicit task performance. Findings from graph-based analyses of such data do suggest changes in the interaction of the CCN and the DN over development (Fair et al., 2008, Power et al., 2010). Early in development, neither network is in an adult configuration, but the constituent regions are also not isolated fragments of their respective adult systems. Instead, communities are organized by anatomical proximity, creating a different inter-network structure in children than adults. The CCN and the DN emerge over time as long-range within-network connections strengthen and anatomically proximal, between-network connections weaken (Betzel et al., 2014, Fair et al., 2008, Power et al., 2010). Taken together, the findings again suggest that developmental change is topological in nature, in this case consisting of the emergence of two distinct networks of opposing function.

### 2.3 Summary and interpretation

In summary, task-based and resting-state fMRI studies provide a seemingly convergent picture of the CCN as:

(a) a highly reproducible feature of the human connectome characterized by positive connections among frontal, parietal, cingulate, and insular cortices and negative connections with DN regions;
(b) a network linked to volitional engagement with the external environment and the suppression of autobiographical reverie and self-reflection; and
(c) a network that changes topologically over development with decreases and increases in the strength of short-distance and long-distance connections, respectively.

The link between cognitive control and development is typically explained in one of three, not mutually exclusive, ways. One possibility stresses the relationship between FC and white matter fiber tracts that connect cortical regions and form the skeleton on which neural activity unfolds. Here, a change in the strength of correlation between two regions over development is explained in terms of the various additive (e.g., myelination) and subtractive (e.g., pruning) processes that occur over the same age range. Network simulations have supported this perspective, showing that FC dynamics of a network, at least as assessed over long-windows of neural activity, largely overlap with the underlying structural skeleton of the network (Honey et al., 2007, Honey et al., 2009). Thus, the persistence of the CCN as a feature of functional data might be the consequence of underlying anatomical structure. SC alone however, may not fully account for FC as two regions can be functionally connected even if they are not structurally connected (Adachi et al., 2012, Buckner et al., 2011). The incongruence of FC and SC has led to a second “integration through synchronization” hypothesis (Fair et al., 2007). This approach argues that ongoing endogenous or task-induced synchronization of activity between two distal regions leads to a strengthening of the FC between the regions via a Hebbian-like learning mechanism that is fine-tuned over development. A third possibility links the emergence of the CCN to competitive interactions with other networks, most notably the DN (Luna et al., 2010). On this account, networks linked to cognitive control and self-referential thought become increasingly independent over development, allowing children to more robustly suppress internally directed thoughts and focus on external goals and actions.

## 3 Caveats of current interpretations

There is abundant evidence that the CCN is a stable feature of the human connectome, linked to cognitive control, and subject to topological change over development. This picture of functional brain organization is however based on several assumptions that falter under close scrutiny, including suppositions about the nature of rsFC, the relation of FC to SC, and the convergence of resting-state and task-based characterizations of the CCN. The current picture of the CCN may prove to be nothing more than just that – a picture.

Consider the reliability of rsFC measures that form the basis of graphical models of the CCN. At long time scales (i.e., 10-min), test–retest reliability is moderate (*r* = 0.39 to *r* = 0.61) (Honey et al., 2009), and is lower for higher-order associative regions that comprise the CCN than for lower-order sensory regions. Honey and colleagues (2009) also noted that reliability is lower than would be expected within a single scan run, even when considering sample size, acquisition noise, or registration artifacts. Variability at short time scales (<1-min) exhibits substantial power in very low frequencies, is lowest between regions with direct structural connections, and is observed in both empirical and simulated resting-state time series. If the very measures that determine network topology are inherently unreliable or unstable, then the structure of a network cannot be characterized as static.

Second, while the structural anatomy constrains interactions between different brain regions and shapes ongoing information processing (Greicius et al., 2009, Hagmann et al., 2008, Hermundstad et al., 2013, Honey et al., 2007, Honey et al., 2009, Shen et al., 2012, Sporns et al., 2007, van den Heuvel et al., 2009, Vincent et al., 2007) (for review, see Damoiseaux and Greicius, 2009) – the relationship between FC and SC is by no means straightforward. Although large-scale structural connections are fixed, at least for the duration of a typical imaging session, functional connections are selectively transformed by specific task demands, intrinsically vary on relatively short timescales, and can deviate substantially from the known SC architecture. FC is thus constrained by, but cannot be wholly predicted, from SC (Honey et al., 2007, Honey et al., 2009).

Finally, there are questions concerning cognitive interpretations that are routinely assigned to resting-state networks. Because profiles of activity that correlate with the instantiation of cognitive control (i.e., task-based maps of the CCN) appear convergent with maps of the CCN generated from resting state data, it is generally assumed that task-based and resting state methods image identical networks. Direct comparisons however, reveal not only that the topology of the CCN differs across task and rest, but also that task-induced topological features are a stronger predictor of behavior than topology assessed in the absence of an overt task (Dwyer et al., 2014). Whether cognitive interpretations of RSNs, such as the CCN, can be upheld is difficult to say for certain. However, to the extent time course correlations within the CCN are evident in the absence of goal-directed thought (i.e., during sleep and anesthesia), they may be a necessary, but are certainly not a sufficient basis for the instantiation of cognitive control.

What is needed then is a model and analysis approach that better approximates the active and time-varying unfolding of cognitive control processes that occurs in real-time. Here, we point to FC dynamics as a possible window through which to explore patterns of brain connectivity that dynamically vary on the time scale of cognitive control, and modifications to these temporal features that occur on the time scale of development.

## 4 Functional connectivity dynamics

### 4.1 What is dynamic functional connectivity?

Dynamic FC is a new framework for understanding brain function that places intrinsic temporal variability of inter-regional coupling at the center of computational theory and empirical methods. Core theoretical ideas draw heavily on insights from the fields of neuroanatomy and electrophysiology and are formally instantiated in models that numerically simulate known functional and structural properties of the brain (for review see Deco et al., 2011). In these models, brain regions are simulated as oscillators – populations of neurons that fire synchronously at neurophysiologically plausible frequencies. Structural connections link simulated brain regions (or local oscillators) allowing local oscillatory activity to propagate and influence synchronous firing patterns in other locales (i.e., allow spatially segregated brain regions to functionally interact). Connections are parameterized to mirror the structural skeleton of the real brain, so that path length, transmission delay, and signal integrity vary as a function of the physical proximity of each coupling pair. Coupling dynamics in these models is therefore highly complex. Even at rest, networks continuously transition between distinct metastable states, or recurring patterns of inter-regional connectivity that fall well outside the natural equilibrium of the system. Importantly, the spatiotemporal characteristics of metastable states are constrained, but not determined by structural connectivity. Connectivity patterns and their temporal dynamics are thus emergent properties of highly constrained and highly inter-active systems.

These models provide a framework for understanding foundational problems in imaging neuroscience, such as the origins of low-frequency BOLD signal fluctuations, the origins of anti-correlated BOLD time courses (such as CCN and DN time courses), and the inherent unreliability of FC measures. Importantly, they have also motivated imaging scientists to revisit assumptions underlying traditional approaches to characterizing functional brain networks (Hutchison et al., 2013a) and evolve new analytic strategies that admit the fundamentally dynamic nature of FC (Allen et al., 2014, Chang and Glover, 2010, Handwerker et al., 2012, Kiviniemi et al., 2011, Sakoglu et al., 2010). Preliminary findings suggest FC fluctuations are linked to underlying neuronal activity (Tagliazucchi et al., 2012) and form meta-stable state patterns (referred to as FC states) that dissolve and reoccur over time (Allen et al., 2014, Liu and Duyn, 2013). These short-lived connectivity patterns can be identified across multiple subjects and deviate substantially from connectivity patterns revealed by traditional approaches.

Consistent with the idea that FC states are a hallmark of complex neural system, they are not exclusive to humans (Hutchison et al., 2013b, Hutchison et al., 2014, Keilholz et al., 2013, Majeed et al., 2011) and are linked to the underlying structural skeleton in that FC stability is dependent on features of the structural topology (Shen et al., 2015a, Shen et al., 2015b). Changes in the temporal features of states have been found during anesthesia-induced unconsciousness (Barttfeld et al., 2015, Hutchison et al., 2014), drug-induced psychedelic experience (Tagliazucchi et al., 2014), and various brain disorders including psychosis (Damaraju et al., 2014, Rashid et al., 2014), epilepsy (Liao et al., 2014), and Alzheimer's disease (Jones et al., 2012). While the approach is still in its relative infancy, results point toward the FC state repertoire and its temporal properties (expression, dwell time, ordering, etc.) as a critical element in normal brain processing. It is possible then that a continuous cycling of brain states underlies the flexibility and power of perception, cognition, and behavior.

Although FC is often used synonymously with resting-state, functional networks can be derived using similar analysis approaches applied to data collected during task-performance (e.g., Krienen et al., 2014), FC can change in association with task demands (Esposito et al., 2006, Fornito et al., 2012, Fransson, 2006, Sun et al., 2007), and tasks can induce time-locked synchronization between regions. Standard GLM analysis yields a static picture of activity in a fixed network of regions. While it is generally accepted that different cognitive tasks engage multiple, possibly overlapping, brain regions, neither the temporal evolution of these patterns, nor their variability between blocks of trials is considered. A cognitive process is thus associated with a single image of an activated region or network of regions. Like resting-state analysis, this oversimplification affords an improved SNR and more straightforward analyses and interpretations, but comes at the cost of a more accurate representation of the processes under investigation.

### 4.2 Dynamic functional connectivity, cognitive control, and development

If brain function is rooted in the dynamics of inter-regional connectivity, how might specific functions, such as cognitive control, be linked to emergent brain dynamics? How might developmental changes in cognitive control be linked to changes in brain dynamics that occur as children mature? And what transformations in the brain – structural or otherwise – cause a shift in brain dynamics over development?

#### 4.2.1 Cognitive control and development

Cognitive control deals with exceptions, or computational challenges for which there are no ready-made solutions, guiding stimulus and motor selection in the face of these unexpected challenges. Young children often respond to unexpected challenges by emitting highly stereotyped – or perseverative – behaviors that were once effective but are now inappropriate based on unanticipated or unacknowledged changes in the environment. Older children and adults, by contrast, respond flexibly to unexpected challenges by considering alternative configurations of a problem space before selecting a particular course of action (Morton and Munakata, 2002, Morton et al., 2003, Munakata, 1998). From the current perspective, developmental changes of this kind should be reflected in age-related differences in the dynamics of FC state transitions, specifically when participants face unexpected cognitive challenges.

**Fig. 2:**
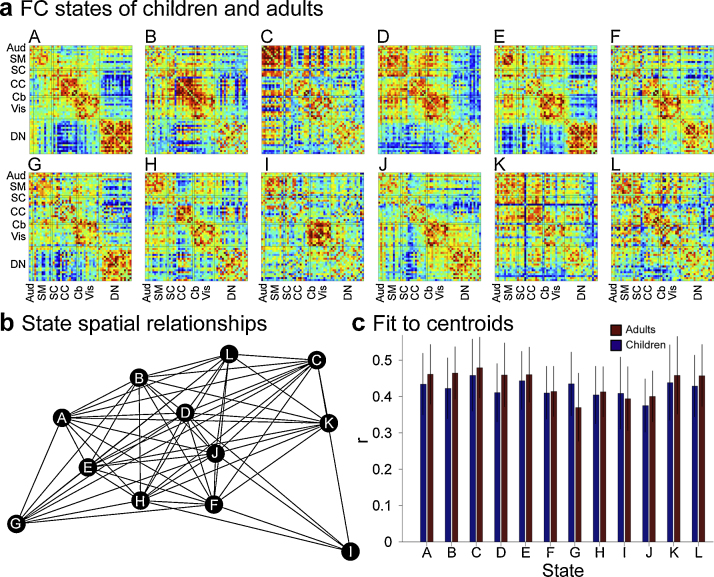
Functional connectivity (FC) states. (a) FC state patterns (A–L) derived from dFC analysis (see text for details) of resting-state and cognitive control condition in both children and adults. (b) A spring-loaded graph representing the spatial correlation of the 12 state patterns with states more similar to each other displayed closer together and more dissimilar displayed further apart. (c) Average state fit of children (<216 months) and adults (≥216 months). Bars represent the mean spatial correlation of patterns assigned to that state with the centroid pattern to which it was assigned, derived separately for children (blue) and adults (red). Error bars represent 1 SD.

Our findings to date support this prediction (Hutchison and Morton, 2015). Participants ranging in age from 9- to 32-years were scanned at rest and during the administration of a challenging cognitive control task. By applying a sliding-window technique to ICA-derived time courses, we measured 750 whole-brain connectivity states from each participant, including 150 and 600 from rest and task data, respectively. Following the method of Allen et al. (2014), k-means clustering was applied to partition windowed states into group-wise FC states. We were able to show that each individual FC state was not a unique (i.e., random) deviation from one overall pattern, but an instance of 1 of 12 recurring patterns (Fig. 2a), each topologically distinct from each other (Fig. 2b). The 12-state repertoire was expressed by participants of all ages, and there were no differences between child and adult exemplars for any of the 12 states (Fig. 2c). Where we did observe age-related differences was in the dynamics of state transitions during the administration of the cognitive control task, with the number of states expressed increasing as a function of participant age. Importantly, this effect was specific to the task condition: during rest, age was unrelated to the number of states expressed.

Data and figures adapted from Hutchison and Morton (2015).

Although preliminary, the findings have several important implications for understanding cognitive control and its development. First, our fMRI-based findings are in agreement with previous electrophysiological evidence (McIntosh et al., 2008) that the complexity of task-evoked brain dynamics increases as a function of age. Divergent methods thus converge on the idea that changes in the complexity of inter-regional functional coupling dynamics is a fundamental feature of brain maturation. Second, we found age-related differences in dynamic transitions between functional connectivity states, but no topological differences between states comprising the state repertoire, a clear point of contrast between the predictions of the current dynamics perspective and that of previous accounts. Computing FC as an average over many time points leads to a static picture of network topology in which individual connections can change in strength with participant age. Averaging in this way however, obscures complex spatio-temporal dynamics and misrepresents differences in FC variability as differences in FC strength. By contrast, analytical strategies that admit dynamic variability in FC reveal a high degree of topological similarity across ages, but differences in whole-brain connectivity state dynamics. Third, our findings highlight differences in what resting-state and task data reveal about developing brain function. While seed-based and graph theoretical rsFC analyses have profoundly advanced the understanding of developing brain function, our findings suggest comparing brain dynamics across task and rest data may be more revealing of age differences in connectivity dynamics than focusing on rest data alone. To the extent that dynamic state transitions reflect an active exploration of alternative functional configurations and are a foundational part of neural computation, differences in brain dynamics across rest and task should be expected and explored (see also Dwyer et al., 2014).

Finally, evidence from our dFC analysis suggests that the instantiation of control is more complicated than selective engagement of the CCN and the suppression of the DN. Of the 12 recurring connectivity states, there were two whose frequency of expression was closely tied to task context (i.e., rest versus task), at least for older participants. One state was marked by strong positive connectivity among CCN, but also selected visual and DN regions (Fig. 2b, state B); a second by disintegration of DN and integration of somato-motor regions with weak connectivity of subcortical and somato-motor regions (Fig. 2b, state C). Clearly demarcated boundaries between “cognitive control” and “default-mode” networks evident over long time scales disappear on shorter time scales, and are replaced by highly fluid configurations that do not obey boundaries spelled out by traditional functional parcellations.

These dFC-based findings are not without obvious limitations and many open questions remain (Box 1). It is unclear whether dynamic changes observed during the task are specific to cognitive control per se or simply task performance more generally. Nor is it clear whether observed age-differences are genuinely developmental or simply a reflection of age-correlated performance differences. However, at a minimum, our findings highlight the possibility that traditional analytic approaches obscure important features of functional brain development.

Box 1 **Open questions and future directions.**

• Are there specific sequences of FC state expression associated with cognitive control?
• Do individual differences in cognitive control predict differences in FC dynamics?
• How early in development does the complete state repertoire emerge?
• What aspects of structural brain development predict changes in FC dynamics?
• Are brain dynamics altered in atypical developmental?

#### 4.2.2 Sources of developmental change

Understanding why whole-brain FC dynamics change over development is an important theoretical and empirical frontier. At present, numerical simulations suggest coupling dynamics within complex systems, such as the brain, are constrained by structural parameters such as path length, transmission time delays, and noise (for review, see Deco et al., 2011). These constraints change with age owing to alterations in brain volume (Cowan et al., 1984, Giedd et al., 1999), myelination (Asato et al., 2010, Yakovlev and Lecours, 1967), neurotransmitter release, and receptor density (Andersen, 2003, Brenhouse and Andersen, 2011, Huttenlocher, 1979), potentially influencing the nature and complexity of whole-brain coupling dynamics over development. To date, empirical tests of these ideas have focused largely on physiological noise and its relation to the complexity of evoked brain dynamics. Electrophysiological investigations for example have shown that the brain not only becomes intrinsically noisier over development, but also exhibits more complex evoked dynamics (McIntosh et al., 2008). Noise may contribute computational capacity by facilitating transitions between different multistable connectivity states given external stimulation. Findings from our own research appear to parallel these ideas, at least indirectly. At rest, inter-regional coupling variability was typically higher among older than younger participants, and brain dynamics were of comparable complexity. However, with the administration of a cognitive control task, there was a marked reversal of these effects. Coupling variability was typically lower among older participants, and brain dynamics were decidedly more complex – the number of states expressed, the number of transitions occurring between multistable connectivity states, and the rate of transition between states were all higher among older than younger participants. Although our understanding – let alone characterization – of developmental changes in FC dynamics remains highly provisional, findings to date suggest important connections between neurophysiological noise, dynamical complexity, and computational function.

### 4.3 Confounding sources of change

There is a trade-off when considering FC dynamics – by increasing the temporal resolution of the analysis (e.g., shortening the sliding window period) there is an accompanying decrease in the number of samples included. While dependent on the methodological approach employed, most estimates of time-varying connectivity patterns rely on several orders of magnitude fewer time points as compared with standard analysis strategies. This leads to an increased susceptibility to noise contamination from hardware (e.g., scanner drift), subject (e.g., motion), and physiological (e.g., variations in respiratory volume/rate and cardiac rate) sources. The risk is that these noise sources can mask more subtle changes that occur over time, or worse, be interpreted as meaningful dynamic variations (see Hutchison et al., 2013a, Hutchison et al., 2013b for greater discussion). This issue is a particular concern in developmental studies due to age-associated motion artifacts (Power et al., 2012; Satterthwaite et al., 2012; Van Dijk et al., 2012; Yan et al., 2013) and normative breathing/cardiac rates. Persistent effects of motion were evident in our initial investigation of age-related changes in FC dynamics across development (Hutchison and Morton, 2015). Although young subjects were initially trained in a mock scanner, all data were subject to standard motion correction and ICA denoising, and subject-wise root mean square motion was included as a nuisance regressor in all models, motion was positively associated with frequency of expression of one of the states (Fig. 2a, State I). While this suggests that motion effects can at least be quantified even if they cannot be eliminated, effects such as micro-movements may not manifest themselves in obvious ways during dFC analysis. A number of techniques have been developed for reducing other noise sources in static approaches (e.g., Beall and Lowe, 2007; Behzadi et al., 2007; Chang and Glover, 2009; Glover et al., 2000; Kundu et al., 2012; Power et al., 2012) though their utility in dynamic analyses is still unclear. Investigators attempting to examine dynamic FC patterns should consider recording respiration and cardiac events with an MRI-compatible pneumatic belt and plethysmograph respectively during data acquisition. That said, the greatest gains in both characterizing and removing noise will likely come from new recording strategies allowing for sub-second whole-brain recordings (Feinberg et al., 2010, Feinberg and Setsompop, 2013).

## 5 Conclusions

Cognitive control is a process whose successful implementation follows a protracted developmental trajectory. Recent results now highlight the critical role that the dynamic expression and tuning of whole-brain, intrinsic temporal coupling patterns plays in this process – challenging the previously held notion that changes in cognitive control are linked to incremental, topological network changes. Many methodological and theoretical questions remain; however, the field is well positioned to explore the link between cognitive control, temporal dynamics, and development.

## Conflict of interest

There are no conflicts of interest to report.
